# Hydrogenated Gold Clusters from Helium Nanodroplets: Cluster Ionization and Affinities for Protons and Hydrogen Molecules

**DOI:** 10.1007/s13361-019-02235-1

**Published:** 2019-06-05

**Authors:** Linnea Lundberg, Paul Martini, Marcelo Goulart, Michael Gatchell, Diethard K. Bohme, Paul Scheier

**Affiliations:** 1 Institut für Ionenphysik und Angewandte Physik, Universität Innsbruck, Technikerstr. 25, A-6020 Innsbruck, Austria; 2 Department of Physics, Stockholm University, 106 91 Stockholm, Sweden; 3 Department of Chemistry, York University, Toronto, Ontario M3J 1P3 Canada

**Keywords:** Hydrogenated gold cluster ions, Helium nanodroplets, Proton affinities, H_2_ affinities

## Abstract

We report the mass spectrometric detection of hydrogenated gold clusters ionized by electron transfer and proton transfer. The cations appear after the pickup of hydrogen molecules and gold atoms by helium nanodroplets (HNDs) near zero K and subsequent exposure to electron impact. We focus on the size distributions of the gold cluster cations and their hydrogen content, the electron energy dependence of the ion yield, patterns of hydrogenated gold cluster cation stability, and the presence of “magic” clusters. Ab initio molecular orbital calculations were performed to provide insight into ionization energies and proton affinities of gold clusters as well as into molecular hydrogen affinities of the ionized and protonated gold cluster cations.

**Electronic supplementary material:**

The online version of this article (10.1007/s13361-019-02235-1) contains supplementary material, which is available to authorized users.

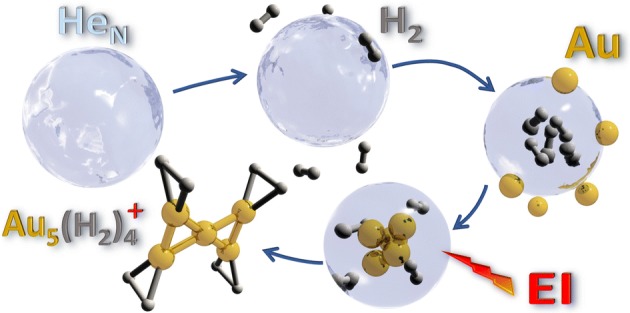

## Introduction

Gold, precious in so many other ways, is at most only moderately effective as a catalyst, at least as a clean bulk metal, when compared to group VIII to X metals including platinum for example, its neighbor on the periodic table. As a hydrogenation catalyst, pure bulk gold has been found to have only a weak affinity for molecular hydrogen (unless dispersed and supported on a metal oxide) [1, 2]. Also, there appears to be no direct evidence that molecular hydrogen chemisorbs by dissociation on bulk gold at room temperature and below.

But gold behaves differently as very small clusters of atoms [3–8]. The chemical nature of small aggregates of gold has been studied extensively in recent decades [9–11] and has led to the development of, for example, gold-based catalysts [12, 13]. More specifically, the history of studies on gold-hydrogen complexes goes back at least a century [14]. Since then, numerous studies of complexes of gold and hydrogen have been carried out [15–17]. Computations have shown that Au_2_ and Au_3_ bind one and even two molecules of hydrogen, the first with binding energies (D_e_) of 0.55 and 0.71 eV, respectively [18]. However, the computations also predict the presence of a substantial energy barrier for the dissociation of adsorbed hydrogen, 1.10 and 0.59 eV, respectively. Other calculations using density functional theory, as well as infrared spectroscopy experiments in solid hydrogen, have characterized AuH, AuH_2_, (H_2_)AuH, and (H_2_)AuH_3_ [19, 20] and the decomposition of AuH_2_ by the release of H_2_ [20]. Sugawara et al. studied reactions of small gold cluster cations Au_n_^+^ (*n* = 1–12) with molecular hydrogen in an FT-ICR mass spectrometer and did not observe any reaction products [21]. However, mixed cluster ions of the form Au_n_H_x_^+^ (*n* < 8) are efficiently formed via laser ablation of a gold rod in an atmosphere of a hydrogen (5.3%)/helium mixture. Pronounced intensity anomalies of these cations as a function of the number of attached hydrogen atoms, *x*, have been reported [21].

Here, we apply a very low temperature technique with which we can encourage both Au atoms to cluster and molecular hydrogen to adsorb on these clusters within a superfluid helium environment provided by helium droplets [22–26]. A beam of nanodroplets of helium is seeded with molecules of hydrogen and atoms of gold and these are allowed to interact before electron impact ionization of the droplets. In this way, clusters of gold and hydrogen are allowed to form and are then exposed to electron and proton transfer reactions that produce positive ions that ultimately are detected mass spectrometrically. The mass spectra provide the stoichiometry of the hydrogenated gold cluster cations as a function of cluster size and, indirectly, insight into the precursor neutral hydrogenated gold clusters. Furthermore, with molecular orbital calculations, we explore the energetics of gold clusters losing electrons or gaining protons as well as the structures and stabilities of the hydrogenated gold cluster cations that are observed to “magically” predominate in the mass spectra.

## Experimental

**Figure 1:**
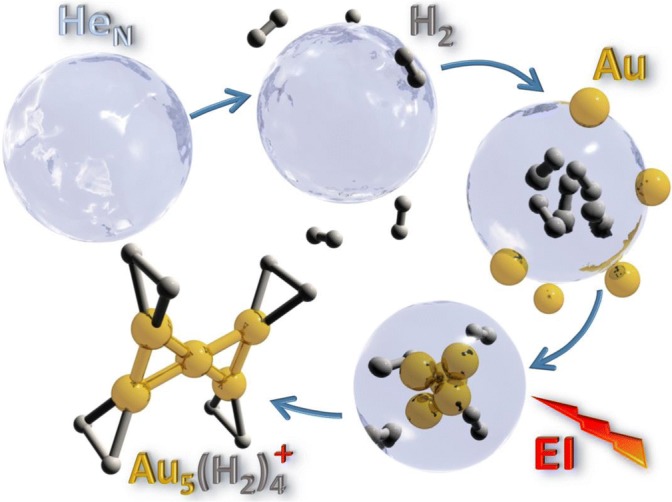
Overview of the formation of gold/hydrogen clusters from doped He nanodroplets. Neutral helium droplets containing millions of atoms capture gas phase H_2_ and Au atoms in sequential pickup chambers and condense into mixed, physisorbed aggregates. The doped droplets are ionized by electron impact (EI) which leads to the formation of charged Au_n_H_x_^+^ clusters that are analyzed by mass spectrometry

The experimental apparatus is described in detail elsewhere [22, 27–29], but an overview of the processes involved can be found in Figure 1. He nanodroplets were produced via supersonic expansion of pre-cooled gaseous He (Messer, 99.9999% purity) under a pressure of 2.25 MPa through a 5-μm diameter nozzle cooled to 9.55 K. The mean size of the produced droplets is estimated to be 10^6^ He atoms [30, 31] and their velocity is approximately 260 m/s. The helium beam passed through a 0.8-mm diameter skimmer and entered a pickup region where hydrogen (Messer Austria GmbH, purity 99.999%) was introduced via a needle valve. Gold vapor was produced from solid gold heated with 118 W, which gives a temperature of at least 950 °C, in an oven similar to the one reported by Feng et al [26] that is located another 115 mm downstream. The vapor pressures in the two pickup cells are on the order of 10^−6^ mbar. The doped droplets underwent ionization in a Nier-type ion source with electron kinetic energies of 85 eV for positive ion formation. The dopants were ionized through different processes depending on the polarity [32, 33]. The ionized complexes were then driven through a set of Einzel lenses into the extraction region of a commercial, reflectron time-of-flight mass spectrometer (Tofwerk AG, model HTOF) where spectra of the signal intensity versus mass per charge were obtained. The spectra were evaluated in the custom software IsotopeFit with which overlaps were deconvoluted, background signals were subtracted, and mass peaks were fitted [34].

## Theory

We have investigated structures and properties of pure and hydrogenated gold clusters with ab initio calculations using second-order Møller-Plesset (MP2) perturbation theory. In order to find the most energetically preferred structure for each cluster size (each unique combination of Au and H atoms), we optimized the structures for any different starting geometries at the MP2/def2-SVP level. We then further optimized the most stable candidates at a higher MP2/def2-TZVP level. Core potentials (as defined in the respective basis sets) were utilized for the Au atoms to include relativistic corrections and to speed up the calculations. A vibrational frequency analysis was performed to ensure that proper minima were achieved in the structure optimizations and to calculate the zero-point energy corrections that are included in the presented energy values. The calculations were performed using the Gaussian 16 software [35].

Molecular ions containing up to 7 Au atoms were studied and, depending on the number of Au atoms, up to 11 H atoms. As the system size increases, so too does the number and complexity of stable isomers as well as the computational resources required. This was the main limitation to the sizes of systems that we have studied. We have investigated a wide range of possible structures for our mixed clusters, including the structures for pure gold clusters from Schooss et al. [27] as initial guesses for the optimizations, and for certain cluster sizes, the preferred structure of the gold atoms changed depending on the number of hydrogens added. A comparison of the pure gold cluster structures and the structures for the magic numbers can be found in the SI.

## Results and Discussion

### Observation of Hydrogenated Gold Cluster Cations

**Figure 2:**
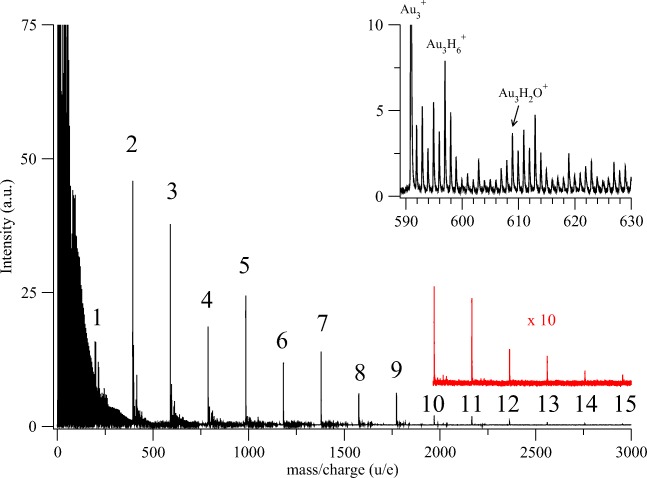
Positive ion mass spectrum obtained by electron ionization of helium nanodroplets showing the pure and mixed gold cluster Au_n_H_x_^+^ peaks. The coarse structure at the lower mass range is composed mainly by pure He clusters. He droplets with a size of about 10^6^ atoms were generated with a nozzle temperature of 9.55 K and a stagnation pressure of 2.25 MPa. They were doped first with H_2_ molecules and then with Au atoms. Pickup pressure was 1.18 × 10^−3^ Pa for H_2_. The metal oven was heated with 118 W. The electron energy for the ionization process was 85 eV. The inset is a close-up on part of the Au_3_^+^ cluster series where the added hydrogens can easily be distinguished. We also see some residual water molecules binding to the gold clusters

Figure 2 shows the intensity distribution obtained mass spectrometrically for the hydrogenated gold cluster cations Au_n_H_x_^+^ seen with *n* up to 15. A clear oscillation in intensity is seen, with odd-numbered clusters generally being more intense than even numbered clusters. With Au_n_^+^ carrying the positive charge, we note that the odd-numbered clusters are even electron systems while the even-numbered clusters are odd electron systems. No doubly charged ions were visible, nor has their observation ever been reported before by others, as far as we are aware.

### Ion Yields

Au doping of HNDs leads to the formation of pure clusters of Au atoms, Au_n_, and when hydrogen is present as well, the formation of hydrogenated clusters Au_n_(H_2_)_m_. The strongly bonded H_2_ molecules are not expected to dissociate in the presence of the gold clusters at the low temperature of the HNDs; the reaction of molecular hydrogen with gold *atoms* to produce AuH is known to be endothermic by more than 1 eV [19].

When the HNDs are exposed to electron impact ionization and He^+^ ions are formed, both Au_n_ and Au_n_(H_2_)_m_ clusters can become ionized by electron transfer to these He^+^ ions (IE[He] = 24.59 eV [36]). We have previously reported the formation of positive clusters of gold atoms under similar conditions in the absence of molecular hydrogen [37]. The excess energy of the electron transfer heats up the charged clusters and can promote its fragmentation. Some cluster ions with a longer lifetime will demonstrate enhanced stability as a consequence of more efficient quenching by the ultra-cold helium matrix. Other precursors of cluster ionization include metastable helium atoms He* as well as He*^−^ or by proton transfer from He_n_H_x_^+^ or H_x_^+^ derived from He^+^ reactions with H_2_ [22] leading to Au_n_H(H_2_)_m_^+^ cations.

**Figure 3:**
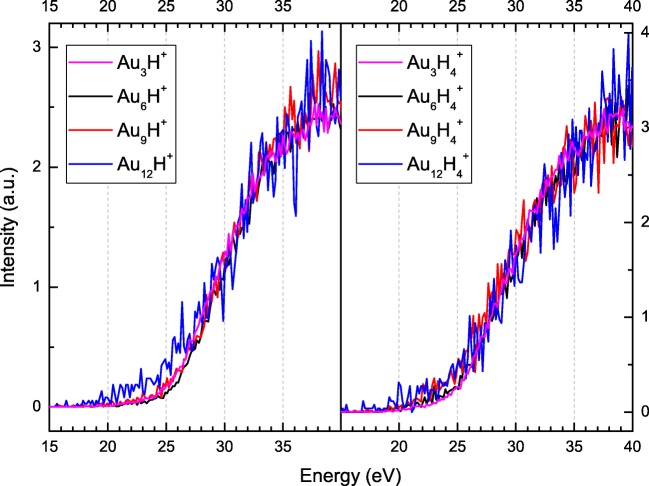
Ion efficiency curves for selected protonated gold clusters, Au_n_H^+^ (left panel), and selected hydrogenated gold cluster cations Au_n_H_x_^+^ (right panel) with *x* = 4 formed upon electron impact on HNDs doped with hydrogen and gold. Helium temperature before expansion was 9.55 K and the pressure 2.25 MPa. The hydrogen pressure was 1.11 × 10^−3^ Pa and the power of the gold oven was 118 W

Figure 3 presents the influence of the energy of the electrons impacting the HNDs, doped with Au and H_2_, on the relative ion yield of protonated cluster ions Au_n_H^+^ with *n* = 3, 6, 9, and 12 and of hydrogenated gold cluster ions Au_n_H_4_^+^ with *n* = 3, 6, 9, and 12. Both populations exhibit an onset around 20 eV, near the 19.8 eV required to form He* in its lowest lying excited state, and there seem to be no remarkable differences in the shapes of the ion profiles. Interestingly, this differs somewhat from the behavior of the ion efficiency curves for pure gold cluster cations, where the position of the maxima shifts towards lower electron energies with increasing cluster size [29]. The reason for this difference is not entirely clear, but could be because of a difference in mean droplet size or by the presence of H_2_ in the droplets.

### Computed Ionization Energies and Proton Affinities of Gold Clusters

Because of the excess energy available in both the electron and proton transfer reactions, Au_n_H(H_2_)_m_^+^ formation can be accompanied by the dissociation of the cluster ions through H_2_ elimination with, as we shall see, the ultimate preferred formation of “magic” hydrogenated clusters of special stability. The excess energy in the ionization of Au_n_(H_2_)_m_ can be considerable because of the high recombination energy of, for example, He^+^ (24.59 eV). Similarly, the very low proton binding energy of the proton donors, e.g., HeH^+^ or H_3_^+^ with PA(He) = 1.82 eV [38] and PA(H_2_) = 4.39 eV [39], leads to high excess energies in the proton transfer to the gold clusters in secondary HeH^+^/H_3_^+^ + Au_n_ → HeH/H_3_ + Au_n_H^+^ reactions that may drive the annealing of the final mixed cluster products.

Ionization energies of small pure gold clusters and their variation with size have been reported previously in the literature, but little appears to be known about the proton affinities and their variation with size. The variation of ionization energy of Au_n_ for *n* = 1–22, known in 2003, has been graphed by Sugawara et al [21]. A striking even/odd oscillation with cluster size *n*, with even-*n* clusters relatively more predominant, is clearly evident and the authors remark on how this oscillation matches that observed in the binding energy D_e_ of Au_n_^+^-Au. The results of our calculations of IE are summarized in Table 1 and plotted in Figure 1. There is agreement as regards both magnitudes and even/odd oscillations in IE. Also included in Table 1 and Figure 1 are our computed values for the proton affinities of the gold clusters. Of note is the sharp increase in PA for clusters with *n* > 1.

**Table 1:** Computed values (∆E_0_ in eV) for the proton affinities, PA, and ionization energies, IE, for gold clusters Au_n_

*n*	1	2	3	4	5	6	7	8
PA(Au_n_)/eV	4.51	7.75	7.89	8.85	8.37	8.07	8.44	8.96
IE(Au_n_)/eV	9.05	10.12	6.63	7.97	7.07	8.89	6.37	7.76

### Observed Profiles of Hydrogenation for Individual Gold Cluster Sizes

Figure 5 provides panels that show the distribution in hydrogenation observed in our experiments for Au_n_H_x_^+^ cluster sizes from *n* = 1 to 8 and *x* from 1 up to 20. These distributions exhibit oscillations and the presence of intense “magic” numbers that shift to higher hydrogenation for clusters with up to 5 gold atoms. Oscillations appear to be more pronounced for odd-numbered gold clusters and at lower degrees of hydrogenation. They are still present for clusters with 6 to 8 gold atoms but strong magic numbers are less pronounced in relative intensity. Another striking feature is the shift from the very pronounced magic numbers that can be seen for *n* ≤ 5 to the richer intensity distributions for *n* ≥ 6. For example, there is a sharp drop in ion yield after Au_6_H_9_^+^, but also rather high intensities of ions with fewer H atoms. This could be an indication of a transition from 2D to 3D structures as the cluster sizes increase, leading to more possible isomers being available, each contributing with their own different magic combinations of Au and H atoms.

We note the following hydrogenation features for specific gold cluster ions:

*n* = *1*: Odd-numbered AuH_x_^+^ are more intense than their even-numbered neighbors with the notable exception of AuH_4_^+^ which clearly exhibits special stability.

*n* = *2*: Au_2_H_5_^+^ clearly predominates and Au_2_H_6_^+^ also has a relatively high intensity compared to all other less remarkable Au_2_H_x_^+^ cluster sizes.

*n* = *3*: The early even-numbered Au_3_H_x_^+^ ions are observed to increase in intensity from *x* = 0 to 2 to 4 to 6. Also for the odd-numbered Au_3_H_x_^+^ ions, an increase can be observed from *x* = 1 to 3 to 5 to 7 with odd *x* Au_3_H_x_^+^ ions being less intense than the preceding even *x* ions. The Au_3_H_6_^+^ ion is the most intense overall.

*n* = *4*: This time, the early Au_4_H_x_^+^ are observed to increase in intensity from *x* = 0 to 7, with a local minimum at *x* = 2 and even numbered ions being less intense than preceding odd *x* ions. The Au_4_H_7_^+^ ion is the most intense overall. Au_4_H_x_^+^ cluster sizes with *x* = 9 to 20 are of low intensity but exhibit a clear odd-even oscillation, with minima at even numbers of H atoms x.

*n* = *5*: As for *n* = 3, the early even-numbered Au_5_H_x_^+^ ions are observed to increase in intensity from *x* = 2 to 4 to 6 to 8 and odd *x* Au_5_H_x_^+^ ions being less intense than neighboring even *x* ions. Au_5_H_8_^+^ and Au_5_H_7_^+^ are the most intense even and odd *x* ions, respectively, with the former being the most intense overall.

*n* = *6*: After the initial appearance of the protonated cluster Au_6_H^+^, oscillations are seen with local maxima of the ion yield of Au_6_H_x_^+^ at *x* = 5 and 8, with Au_6_H_8_^+^ being slightly more predominant. A sharp drop-off in ion intensity is seen after Au_6_H_9_^+^. Curiously, a “rogue” cluster ion Au_6_H_17_^+^ shows a small maximum beyond Au_6_H_11_^+^.

*n* = *7*: Four strong oscillations are seen early on for the even *x* cluster ions Au_7_H_x_^+^ with *x* = 2, 4, 6, and 8 with *x* = 6 predominating. The ion yields for cluster ions Au_7_H_x_^+^ with *x* > 9 exhibit no odd-even oscillation.

**Figure 4:**
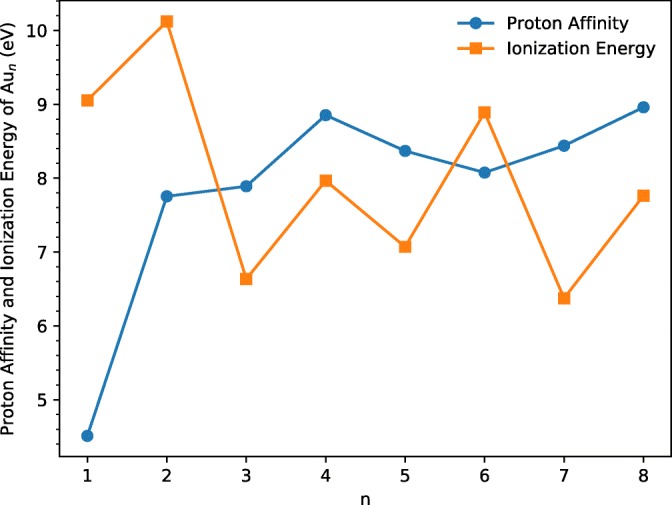
Computed variations in the ionization energy and proton affinity of gold clusters Au_n_ with cluster size n. All values correspond to ∆E_0_

*n* = *8*: A strong protonated cluster peak Au_8_H^+^ is followed by strong adduct peaks with one and two H_2_ molecules. Note from Table 1 and Figure 4 that the calculations indicate that Au_8_ has the highest proton affinity (7.76 eV) of the systems studied here.

**Figure 5:**
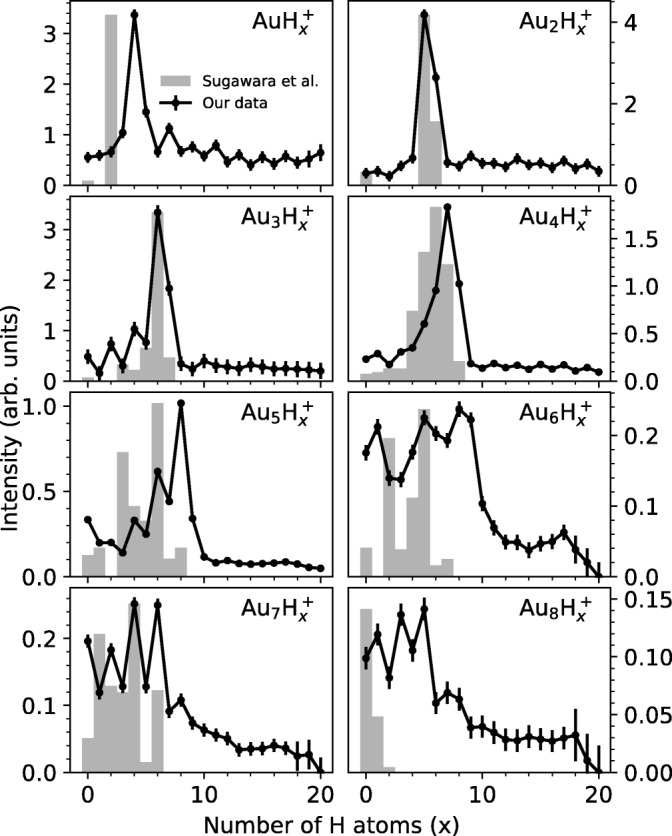
Cluster series of Au_n_H_x_^+^ from *n* = 1 to 8 and *x* from 1 up to 20, extracted from the mass spectrum shown in Figure 1 using the IsotopeFit software [34]. Magic number peaks are clearly visible as are odd-even oscillations corresponding to clusters with H_2_ molecules in the presence or absence of an H atom. The bar peaks represent the FT-ICR data of Sugawara et al. [21]

Figure 5 also includes the FT-ICR data of Sugawara et al. [21] obtained in experiments with the laser ablation of gold in a H_2_(5.3%)/He mixture (gray bars). Hydride gold cluster distributions are observed that are sometimes similar but more often distinctly different from ours. The extent of hydrogenation is generally seen to be much smaller, but the presence of magic number intensities for Au_2_H_5_^+^ and Au_3_H_6_^+^ coincides with ours. Magic numbers in the FT-ICR spectra are also observed otherwise, but generally shifted to lower hydrogenation. These differences may well be due to the higher temperature of the FT-ICR experiments, direct formation of Au_n_^+^ cluster ions by laser ablation, and a significant presence of H atoms in the Au_n_^+^ cluster ion formation region.

### Computed Structures of Hydrogenated Gold Cluster Cations

**Figure 6:**
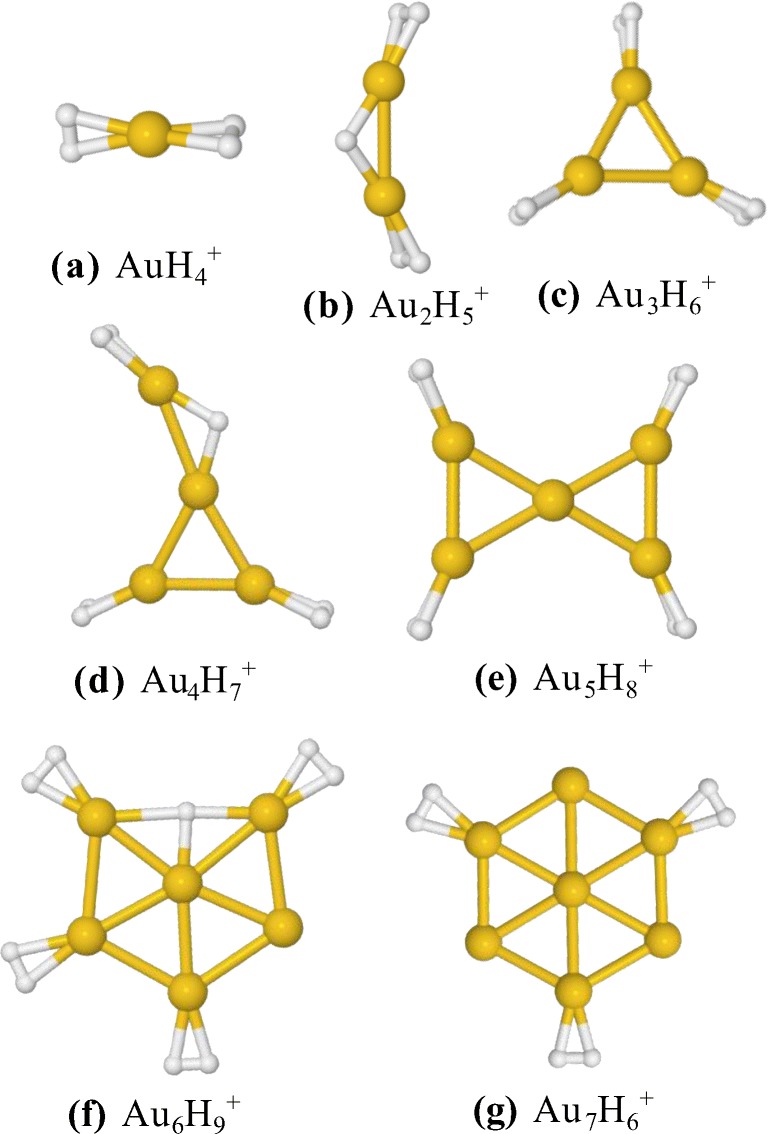
Proposed structures for the most abundant clusters of Au_n_H_x_^+^ for each series of *n* calculated at MP2/def2-TZVP level of theory. All the gold “skeletons” that are shown are planar except Au_7_H_6_^+^ which agrees with the nonplanar Au_7_^+^ structure

Figure 6 displays the structures for some of the most abundant “magic” clusters of Au_n_H_x_^+^ for each series of *n* = 1 to 7 calculated at the MP2/def2-TZVP level of theory. The even-numbered gold cluster cations are open shell radicals and thus preferentially protonated. In general, the protonated gold clusters (Au_n_H^+^) have structures that are very similar to the next larger (closed shell) pure gold cluster cations (Au_n+1_^+^). All the structures that are shown are planar, in regard to the positions of the Au atoms, except Au_7_H_6_^+^, which agrees with the nonplanar Au_7_^+^ structure. Comparisons between the structures in Figure 6 and the bare Au_n_^+^ structures can be found in the SI.

For each combination of Au and H, several structures were optimized to find the one with the lowest potential energy. In the cases of the “magical” structures, alternative isomers found have at least 0.1 eV higher energy than the proposed minima.

The calculations suggest that H_2_ molecules bond directly to Au atoms of the gold cluster “skeleton” and that the extra H atom in the even-numbered gold clusters (*n* = 2, 4, and 6) simply bridges two Au atoms.

### Computed Energies of Hydrogenated Gold Cluster Cations

**Figure 7:**
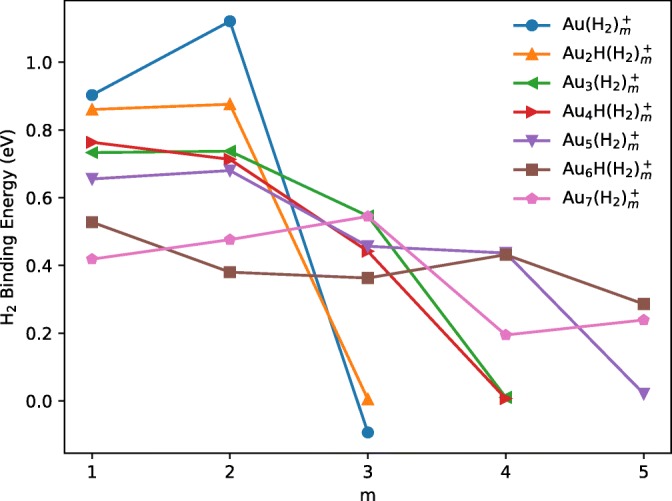
The binding energies of intact H_2_ units in Au_n_(H_2_)_m_^+^ → Au_n_(H_2_)_m-1_^+^ + H_2_ (or Au_n_H(H_2_)_m_^+^ → Au_n_H(H_2_)_m-1_^+^ + H_2_) processes. There is a general decrease in binding energies of H_2_ after the “magic” cluster sizes have been reached and more hydrogens are added to the structures

The odd-even oscillations seen in the data shown in Figure 5 correspond to cluster cations with added intact H_2_ molecules in the presence or absence of an H atom. In our calculations, we explored the H_2_ affinities (∆E_0_) of the gold cluster cations for molecular hydrogen. The results are summarized in Table 2 and graphed in Figure 7. Hydrogenation with H_2_ molecules was seen to be limited with larger cluster cations exhibiting a greater capacity for hydrogenation but weaker bonding of individual hydrogen molecules. Up to two H_2_ molecules bind strongly to Au^+^ and Au_2_H^+^ with energies of 0.8 to 1.1 eV. Au_3_^+^ and Au_4_H^+^ have a significant affinity for up to three molecules of H_2_, the first two with about 0.7 eV and the third somewhat lower still by 0.2 and 0.3 eV, respectively. The H_2_ affinities of Au_5_^+^, Au_6_H^+^, and Au_7_^+^ are the lowest, below 0.68 eV, but the trends suggest that the “magic” numbers seen in the experiments correspond to gold clusters that are saturated with a first layer of relatively strongly bound H_2_ units.

**Table 2:** Binding energies (∆E_0_ in eV) computed for gold cluster cations Au_n_(H_2_)_m_^+^ (odd number of gold atoms) and Au_n_H(H_2_)_m_^+^ (even number of gold atoms) for *m* = 1 to 5. Numbers in bold correspond to “magic” clusters of relatively high intensities

*m*	Au_1_(H_2_)_m_^+^	Au_2_H(H_2_)_m_^+^	Au_3_(H_2_)_m_^+^	Au_4_H(H_2_)_m_^+^	Au_5_(H_2_)_m_^+^	Au_6_H(H_2_)_m_^+^	Au_7_(H_2_)_m_^+^
1	0.90	0.86	0.73	0.76	0.66	0.53	0.42
2	**1.12**	**0.88**	0.74	0.71	0.68	0.38	0.48
3	− 0.09	0.00	**0.55**	**0.44**	0.46	0.36	**0.54**
4			0.01	0.01	**0.44**	**0.43**	0.19
5					0.02	0.29	0.24

## Conclusions

Our experiments have shown that H_2_ molecules readily attach to gold clusters with up to at least 8 gold atoms in a He environment near zero K. These hydrogenated clusters are readily ionized in the presence of electron acceptors such as He^+^ or proton donors such as HeH^+^ and some H_2_ elimination may ensue due to the high excess energy of these processes. There was no evidence for the dissociation of adsorbed H_2_ molecules; there was no indication of H elimination that might result from dissociation. The hydrogenated gold cluster ion distributions exhibit “magic” features that appear to reflect special stabilities for certain numbers of H_2_ adsorbed molecules.

Our calculations have indicated that the number, including the “magic” number of H_2_ adsorbed molecules, is determined by the structure of the underlying (most often flat) Au cluster skeleton and the number of Au atoms exposed on the periphery. The computed H_2_ affinities of the cation clusters are as high as 1.1 eV, but weaken with increasing cluster size. H atoms appear to bridge two Au atoms in hydrogenated clusters with an even number of Au atoms.

## Electronic supplementary material

ESM 1 (DOCX 816 kb)
