# Supplementary material for: Hydrogenated Gold Clusters from Helium Nanodroplets: Cluster Ionization and Affinities for Protons and Hydrogen Molecules
Source: J Am Soc Mass Spectrom. 2019 Jun 5;30(10):1906–13. doi: 10.1007/s13361-019-02235-1 (PMC6805818; doi:10.1007/s13361-019-02235-1)
Supplement: Supplementary file 1 — (DOCX 816 kb) [file 13361_2019_2235_MOESM1_ESM.docx]

**Figure 1**: Proposed structures for the bare gold clusters, Au_n_^+^, where *n* = 3 - 7 compared with the most abundant structures for Au_n_H_x_^+^. The respective gold cluster structures are the same for *n* = 3, 5, 7 and different for *n* = 4, 6. The structures are calculated at MP2/def2-TZVP level of theory. All gold structures are planar except Au_7_H_6_^+^/Au_7_^+^.
